# Are cognitive deficits more severe among people with treatment-resistant schizophrenia than treatment responders?

**DOI:** 10.1177/10398562251391697

**Published:** 2025-10-30

**Authors:** Ananthakrishnan Koloth, Kim Yeak, Somayyeh Azimi, Radhakrishnan Koloth, Aneeka Alexander, Milan Dragovic, Alexander Panickacheril John

**Affiliations:** Sir Charles Gairdner Hospital, 94267 North Metropolitan Health Service, Perth, WA, Australia; 94922 Bentley Health Service, Bentley, WA, Australia; Clinical Research Centre, 56379 North Metropolitan Health Service Mental Health, Perth, WA, Australia; 94923 Peel and Rockingham Kwinana Health Service, Perth, WA, Australia; Northern Beaches Hospital, Sydney, NSW, Australia; Clinical Research Centre, 56379 North Metropolitan Health Service Mental Health, Perth, WA, Australia; 94922 Bentley Health Service, Bentley, WA, Australia; and Medical School, 2720 The University of Western Australia, Perth, WA, Australia

**Keywords:** schizophrenia, treatment resistance, cognition in psychosis, cognitive deficits, clozapine

## Abstract

**Background:**

Given the robust association of cognitive deficits (CD) with community functioning in people with schizophrenia and the inconsistent findings from the limited research on CD among people with treatment-resistant schizophrenia (TRS), we evaluated the neurocognitive functions of patients with TRS.

**Methods:**

We retrospectively collated data on cognition and selected demographic and clinical variables of 181 patients with TRS and 59 with treatment-responsive schizophrenia (non-TRS) admitted to a psychiatric rehabilitation facility from 2010 to 2019. The Brief Assessment of Cognition in Schizophrenia (BACS) was used to evaluate cognitive function. Multivariate analysis was utilised to compare the BACS scores of the two groups.

**Results:**

TRS patients had more severe CD than non-TRS patients, with the BACS composite score (*p* = .003), verbal memory and learning (*p* = .003), working memory (*p* = .043), and psychomotor speed (*p* = .004) significantly worse in the TRS group. There was no significant difference in the BACS scores between the 60% of the TRS patients who were treated with clozapine and the TRS patients (40%) treated with other antipsychotics.

**Conclusion:**

CD are more severe among patients with TRS than in non-TRS cohorts. The pathophysiology of CD among people with TRS and efficacy of interventions to ameliorate CD among this cohort warrant greater scrutiny.

Cognitive deficits (CD) affecting multiple domains are common among people with schizophrenia and are core symptoms at all stages of the disorder.^1,2^ Assessment and management of CD in schizophrenia are of paramount importance in clinical practice given the robust relationship of CD with community functioning, negative and disorganised symptoms, aggressive behaviour, hospitalisation, and high healthcare costs.^1–3^ It has been reported that certain subgroups of patients with schizophrenia, such as those with deficit schizophrenia or comorbid metabolic syndrome or diabetes, could experience greater severity of impaired cognition.^4,5^

Nearly 40% of people with multi-episode schizophrenia and a quarter with first-episode schizophrenia continue to experience moderate or more severe psychotic symptoms despite adequate trials with two or more non-clozapine antipsychotics and are considered as having treatment-resistant schizophrenia (TRS).^6,7^ People with TRS generally have more prominent markers of illness severity, such as a lower quality of life, higher unemployment, poorer achievement of life milestones, more inpatient admissions, higher prevalence of substance abuse, more severe negative symptoms, and significantly higher healthcare costs compared to those people with schizophrenia who respond favourably to treatment.^8,9^ The more severe functional impairments and clinical severity among those with TRS and the strong relationship of these parameters with CD in schizophrenia point towards the possibility that people with TRS could experience more severe CD. The profile of cognitive functions among people with TRS and its comparison with those having treatment-responsive schizophrenia (non-TRS) could offer valuable insights into the pathophysiology of TRS. However, research in this area has received limited attention and there is a paucity of studies comparing the neurocognitive performance of people with TRS and people with schizophrenia who do not meet the criteria for TRS. Furthermore, the limited number of studies in this area have generated inconsistent results, with no significant difference in cognitive function, significantly worse cognitive function across several domains, and worse cognitive function in select cognitive domains reported among people with TRS by different authors.^10–16^ In addition, the sample sizes of these studies, particularly that of the TRS group, were relatively modest, ranging from 19 to a maximum of 53 patients. Given these, we evaluated the neurocognitive functions of patients with TRS, comparing them to those people with schizophrenia who had a favourable response to antipsychotics and did not meet the criteria for TRS.

## Materials and methods

### Participants

This study was conducted at a 12-bed public psychiatric inpatient rehabilitation unit located in Perth, Western Australia. This facility cared for people with severe mental illness and aimed for a median length of stay of around 3 months. It was the referral centre for 6 public mental health services of metropolitan Perth. More than 90% of patients admitted to the unit had a diagnosis of schizophrenia spectrum disorders. Patients were admitted to the unit once their acute symptoms were stabilised for further rehabilitation and placement. Only voluntary patients who were willing to undergo rehabilitation and further treatment were considered for admission. Diagnosis was made using the International Classification of Diseases 10th Revision (World Health Organization, 1992) criteria by a senior consultant psychiatrist, the clinical lead of this unit’s multidisciplinary mental health team. As part of usual clinical practice in the unit, in addition to other clinical assessments, cognitive function of patients was evaluated using a standardised instrument usually within 2 weeks of their admission to the facility. For this study, after obtaining permission from the South Metropolitan Health Service Human Research Ethics Committee, we retrospectively collected from the patient’s medical records relevant data of patients with a diagnosis of schizophrenia or schizoaffective disorder who were admitted to this facility from 2010 to 2019.

### Measurement of cognitive function

The Brief Assessment of Cognition in Schizophrenia (BACS) was used to measure patients’ cognitive function in this unit. The BACS takes approximately 35 min to administer, provides age and gender-standardised z scores, and has demonstrated robust reliability and concurrent validity against lengthy neuropsychological tests.^17^ The BACS consists of 6 subtests: list learning (verbal memory and learning), digit sequencing (working memory), token motor task (motor speed), verbal fluency (semantic fluency), symbol coding (attention and speed of information processing), and Tower of London (executive function- reasoning and problem-solving). The developers of the BACS established normative scores by administering the instrument to 404 healthy controls. The scores were organised in a way that allows the calculation of standardised scores (z-scores) adjusted for age and sex for the six subtests and a composite cognitive score, which is the unweighted average of the scores on the subtests. BACS testing was performed by clinical psychologists or a senior mental health nurse working in the unit after building rapport with the patients and once acute psychotic and disorganisation symptoms were stabilised.

### Treatment resistance

After a comprehensive review of the patient’s medical records, among those with the diagnosis of schizophrenia or schizoaffective disorder, treatment resistance was determined by a senior psychiatrist based on the persistence of at least moderate positive symptoms despite trials with at least two different antipsychotics with a minimum chlorpromazine-equivalent dose of 600 mg/day for a minimum of six consecutive weeks each.^18^ Patients who were managed on clozapine were considered as having TRS unless it was commenced for indications other than treatment-resistant schizophrenia or schizoaffective disorder. Patients with schizophrenia whose psychotic symptoms had responded favourably with mild or less psychotic symptoms on treatment with adequate doses of non-clozapine antipsychotics were classified as having treatment-responsive schizophrenia (non-TRS group).

### Data collection

The collected data included sociodemographic details, such as age, gender, employment status and accommodation, and clinical information, such as principal diagnosis, comorbidities, current substance abuse, duration of psychosis, whether the patient met the criteria for a clinical diagnosis of TRS or has been on treatment with clozapine were collated. In addition, the raw and z-scores of the BACS subtests and composite scores were collected. Duration of psychosis was obtained by subtracting the age at the time of onset of first psychotic symptoms from the age at the time of admission to the facility.

### Inclusion and exclusion criteria

We included in this study consecutive patients admitted with diagnoses of schizophrenia/schizoaffective disorder to the unit who had BACS testing completed on admission. However, patients with diagnoses of intellectual disability or who had neurological disorders such as epilepsy or head injury were excluded.

### Ethics approval

The study was approved by the South Metropolitan Human Research Ethics Committee, Perth, Western Australia (project number RGS0000002167). The data was collected under a waiver of requirement of consent from individual patients approved by the HREC.

### Statistical analyses

We divided the patients with diagnoses of schizophrenia and schizoaffective disorders into two groups: those with TRS and non-TRS. All data analysis was performed using the Jamovi software package (version 2.2.5). Descriptive statistics were used to describe the characteristics of the TRS and non-TRS groups. Multivariate analysis of covariance (MANCOVA) and analysis of covariance (ANCOVA) was utilised to compare the standardised z-scores of six subtests and the composite score of the BACS.

## Results

There were 322 admissions of people with severe mental illnesses to the unit during the study period. Of these, 240 patients with schizophrenia or schizoaffective disorder had BACS completed on admission. Of these, 218 (90.8%) had a diagnosis of schizophrenia, and 22 (9.2%) had schizoaffective disorder. One hundred eighty-one participants were classified as having TRS and 59 as non-TRS. Of the 181 patients in the TRS group, a significant proportion (109, 60.2%) were managed with clozapine.

### Demographic and clinical characteristics of the TRS and non-TRS groups

Table 1 displays the collected demographic and clinical characteristics of the 2 groups. The mean age was 2 years more at 33.1 years among people in the TRS group. Males were preponderant in both groups, and 88% of people in the non-TRS and 94% in the TRS group were unemployed. While psychiatric comorbidities and substance abuse were common, there was no significant difference in age, gender, accommodation, occupation, principal psychiatric diagnosis, presence of comorbidities, and substance abuse between the groups. However, those in the TRS group had a significantly longer duration of psychosis compared to the non-TRS group (t = 4.42, *p* < .001).

**Table 1.** Descriptive analysis of treatment-resistant and non-treatment-resistant schizophrenia patients.

Variables	Schizophrenia type		Test	*p*-value
	Non TRS *n* = 59	TRS *n* = 181		
Gender – *n* (%)				
Male	46 (78.0)	124 (68.5)	1.93^a^	0.165
Female	13 (22.0)	57 (31.5)		
Age – M (SD)	31.1 (10.2)	33.1 (10.5)	−1.31^$^	0.190
Employment				
Competitive employment	4 (7.0)	6 (3.4)	2.83^a^	0.243
Non-competitive work	3 (5.3)	4 (2.3)		
Unemployed	50 (87.7)	167 (94.4)		
Accommodation				
Independent head of household	7 (11.9)	37 (20.6)	6.46^a^	0.167
Semi-independent head of household	1 (1.7)	8 (4.4)		
Not head of household	14 (23.7)	34 (18.9)		
Residential care/fully supported	15 (24.5)	57 (31.7)		
Homeless	22 (37.3)	44 (24.4)		
Diagnosis -*n* (%)				
Schizophrenia	52 (88.1)	166 (91.7)	0.684^a^	0.408
Schizoaffective disorder	7 (11.9)	15 (8.3)		
Duration of psychosis (year) – M (SD)	4.96 (6.25)	9.65 (7.30)	−4.42^$^	**<0.001**
Presence of comorbidity – *n* (%)	42 (71.2)	143 (79.0)	1.54^a^	0.215
Substance abuse -*n* (%)				
Alcohol	23 (39.0)	74 (40.9)	0.07^a^	0.796
Stimulants	15 (25.4)	54 (30.0)	0.45^a^	0.501
Cannabis	25 (42.4)	84 (46.7)	0.33^a^	0.566
Other drugs	8 (13.8)	23 (13.1)	0.02^a^	0.888

^a^Chi-square test, $ t-test.

Bold fonts that the p values are signficant.

### Comparison of cognitive function (BACS scores) between the TRS and non-TRS groups

Results of multivariate analysis of variance, which included all BACS subtests z-scores, indicated statistically significant differences in cognition in the TRS group compared to the non-TRS group when controlling for the effects duration of psychosis (F = 2.23, *p* = .042, Cohen’s d = 0.49). Subsequent univariate analysis revealed significant differences in verbal memory and learning, digit sequencing, and token motor task scores, with worse scores among the people in the TRS group (Table 2). However, verbal fluency, symbol coding and Tower of London tests, while more impaired in the TRS groups, was not significantly different between the groups.

**Table 2.** Univariate analysis of variance of the 6 subtests of BACS of treatment-resistant and non-treatment-resistant schizophrenia patients.

Variables	TRS	Non-TRS	F	*p*-value	Cohen’s d
	Mean (SD)	Mean (SD)			
Verbal memory & learning	−1.81 (1.4)	−1.09 (1.3)	8.85	**0.003**	0.40
Digit sequencing	−1.35 (1.1)	−0.95 (1.3)	4.13	**0.043**	0.27
Token motor task	−0.81 (1.2)	−0.24 (1.3)	8.27	**0.004**	0.39
Verbal fluency	−0.94 (1.2)	−0.67 (1.2)	1.04	0.310	0.14
Symbol coding	−2.08 (1.0)	−1.75 (1.1)	3.43	0.065	0.25
Tower of London	−0.38 (1.4)	−0.06 (1.0)	1.82	0.179	0.18

Bold fonts that the p values are signficant.

The effect sizes of the difference in the BACS subtests between the TRS and non-TRS groups were small, ranging from 0.14 (verbal fluency) to 0.4 (verbal memory and learning). Additionally, the analysis of covariance of the composite score revealed significant differences in cognition between the TRS and non-TRS groups after controlling for the effects of duration of psychosis mean (z-score difference = 0.68, F = 9.33, *p* = .003, Cohen’s d = 0.40).

**Figure 1.**
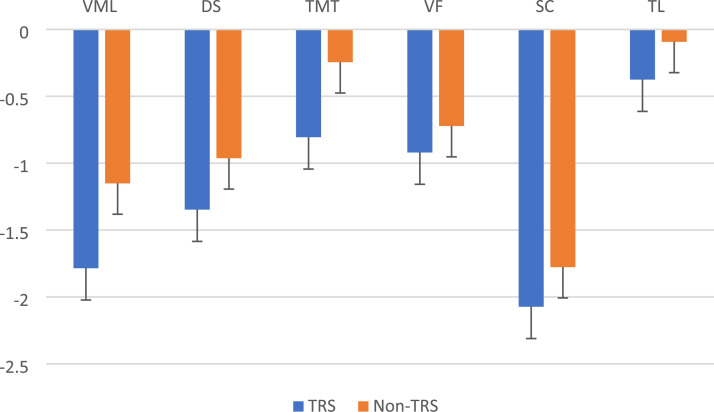
Estimated marginal means of BACS subtests in the TRS group compared to the non-TRS group. VML: Verbal memory and learning; DS: digit sequencing; TMT: token motor task; VF: verbal fluency; SC: symbol coding; TL: Tower of London.

Figure 1 illustrates the estimated marginal means (z-scores) in the BACS for each subtest between the TRS and non-TRS groups. Processing speed (symbol coding) was the domain most affected in both the TRS and non-TRS groups, followed by verbal memory and learning and digit sequencing. The Tower of London test was the least affected. The results also showed a higher difference in the mean of verbal memory and learning (0.63) and TMT (0.56) scores between the TRS and non-TRS groups (Figure 1).

### Influence of clozapine

We separately examined the subgroup of TRS patients receiving clozapine and compared their BACS z scores to those of TRS patients not treated with clozapine. The effect of duration of psychosis was controlled, and the results indicated no significant difference in cognition among TRS patients who were treated with clozapine compared to those not treated with clozapine (F = 0.72, *p* = .636).

## Discussion

### CD in TRS

Utilising a reasonably large sample size of patients and standardised cognitive function examination, we found that people with TRS had significantly worse cognitive function than treatment-responsive patients with schizophrenia, both in overall cognition and subdomains such as verbal memory, working memory, and psychomotor speed. Previous studies that evaluated and compared general cognition and subdomains of cognition among people with TRS and non-TRS are sparse, had small sample sizes for the TRS group, and have generated heterogeneous findings.^10–16^ While some studies did not find a significant difference between the two groups,^10^ others observed widespread neuropsychological deficits among people with TRS compared to the non-TRS population.^13,15^ Yet others have reported more significant deficits in selected cognitive domains such as verbal memory and learning,^12,14^ working memory,^14^ verbal intelligence and fluency,^16^ sustained attention,^12^ and visuospatial function and visual memory,^11^ among the TRS group. A recent meta-analysis of the limited number of studies on cognition among people with TRS observed that that the available studies together evaluated only 154 to 603 people on the various subdomains of cognition such as attention, working memory and processing speed, verbal memory and learning, visual−spatial memory and learning and executive function.^19^ They reported that the cognitive scores of TRS patients were lower than treatment-responsive patients, with the effect sizes ranging from small to moderate, and the largest effect size was for verbal memory and learning (d = −0.53). They also observed that these effect sizes were statistically significantly different from 0, except for executive function and visual−spatial memory and learning. However, the results of individual studies on executive functions had some degree of heterogeneity.^19^ Our results closely mirror their finding that verbal memory and learning, processing speed, working memory, attention and general cognition were significantly worse among the TRS group but not executive function and visual-spatial memory. An umbrella review of the systematic reviews and meta-analyses of cognitive deficits in people with schizophrenia found that while most cognitive domains are more affected in people with schizophrenia compared to both healthy controls and people with mood disorders, the differences were more pronounced in the domains of processing speed, verbal memory, and working memory and these profile of impairment was stable across the course of the illness.^1^ While the reasons for disproportionate affection of verbal memory and processing speed in schizophrenia and TRS compared to executive functions is not clearly elucidated, this pattern could be due to a combination of neurobiological and developmental factors.^16,19–21^ Methodological differences in the selection of TRS and non-TRS patients, differing instruments used to assess cognitive functions, confounding differences in the treatment received, and other sociodemographic factors and illness-related parameters could also account for some of the variations in the findings in different studies.^10–16,19^

A significant relationship between suboptimal community function, such as poorer employment outcomes, living situation, and skill acquisition and cognitive impairments, has been demonstrated in people with schizophrenia.^1–3,22^ It could be postulated that the greater severity of impaired community functioning and psychosocial care needs found among people with TRS,^8,9^ could at least in part, be attributed to the greater severity of CD observed among them. Furthermore, the more significant impairment in verbal memory and working memory found in our study and some other studies has relevance for psychiatric rehabilitation in the context of the findings that verbal memory was a better predictor of employment outcomes and community status than other clinical symptoms among patients with TRS.^22,23^

The pathophysiology of CD in schizophrenia and the reasons for increased CD observed among people with TRS are not clearly understood.^24^ Interestingly, the effect sizes of the greater CD observed among people with TRS compared to people with treatment-responsive schizophrenia in our study and in the metanalysis by Millgate et al.^19^ are broadly similar in magnitude to the effect size of the reported differences in neurocognitive functions between unaffected first-degree relatives of people with schizophrenia and healthy controls.^25^ These lend some support to the hypothesis that TRS lies in a more extreme position on a continuum of neurodevelopmental and genetic vulnerability for schizophrenia.^16,19^ However, in view of the nonresponse to traditional antipsychotics from illness onset among most people with TRS, other researchers have postulated that TRS is a categorically distinct type of schizophrenia without hyperdopaminergia, but with changes in receptor signalling of glutamate or other neurochemicals such as serotonin or GABA, and or other biological systems such as oxidative stress pathways or lipid peroxidation, distinctive neuroimaging findings and greater genetic loading and neurocognitive impairments.^20,21^ It can also be speculated whether some of the distinct biological changes observed in TRS, such as aberrant glutamatergic and dopaminergic functions, reductions in grey matter and reduced perfusion of frontotemporal and hippocampal regions, and disruption of white matter connectivity, are relevant for the pathophysiology of increased CD observed in this condition.^20,21,24^

Reviews and meta-analyses on the effect of clozapine on cognition in patients with schizophrenia have reported inconsistent findings. A recently published metanalysis of studies of patients with TRS who were treated with clozapine who had baseline and follow-up cognitive functions assessments revealed improvement in some cognitive domains such as symbol coding and verbal fluency with small effect sizes, particularly among young cohorts and those who were on clozapine for more than 6 months.^26^ In our cross-sectional study, we did not find significant difference in the CD among people with TRS who were treated with clozapine or other antipsychotics. However, we did not ascertain their cognitive function of these people prior to the commencement of clozapine or other antipsychotics and hence cannot comment whether treatment with clozapine had positive, negative or no effect on cognition in our cohort of patients. We also did not calculate the duration the patients were treated with clozapine before undergoing the cognitive testing. The inconsistent findings in the literature call for further longitudinal studies on the changes in cognition and functioning among people with TRS who are consistently treated with clozapine for a length of time.

Given the robust association between functional impairment and illness severity with CD in schizophrenia, and the more severe CD observed in people with TRS, effectively treating CD in TRS is essential. While the results from the meta-analysis of longitudinal studies of clozapine use among people with TRS are encouraging, clozapine is under-prescribed for people with TRS.^27^ There is no convincing evidence for the efficacy of non-clozapine antipsychotics for CD in TRS.^1,2^ Treatments such as cognitive remediation, exercise interventions and combining these treatments with other cognition-enhancing pharmacological and non-pharmacological treatments have been found moderately beneficial for cognition and functioning in people with schizophrenia.^28–30^ Such studies are lacking in TRS, and the efficacy of these interventions must be systematically trialled among people with TRS.

### Strengths and limitations

The study has notable strengths, such as using a standardised instrument for cognitive function assessment and the cognitive evaluation done by experienced professionals working in the unit after building rapport with patients. Data was meticulously collected by a senior psychiatrist and experienced mental health clinicians working in the unit. The sample size of the TRS group (181) is significant and among the highest in this field of research. However, this study was retrospective and involved collecting documented data from patients’ medical records. While we found the quality of documentation in the tertiary care unit to be good, the accuracy of the data could not be cross-checked through direct patient interviews. While documentation and extraction of information bias cannot be excluded, the data was collected after a comprehensive review of patients’ medical records. Given the specialised nature of the study setting as a referral centre and the high proportion of those with TRS in our sample, extrapolating these findings to other settings cannot be guaranteed. Furthermore, we did not evaluate the impact of the positive, disorganised and negative of schizophrenia on cognitive symptoms and their assessment. However, cognitive function assessments were done once the psychotic symptoms were stabilised.

In conclusion, our study demonstrated that CD affecting multiple domains were prominent in people with TRS, and global cognition, verbal memory, and working memory were more severely affected among people with TRS compared to non-TRS cohorts. Given the relevance of CD for functional impairment and other illness-related parameters and the limited research exploring cognition among people with TRS, studies exploring the aetiology and pathophysiology of CD and its treatment among people with TRS are warranted.

## Data Availability

Data related to the study is available on reasonable request to the corresponding author once permission is obtained from the ethics committee.*
